# Feasibility and Safety of a Novel Pig‐tail Inside Stent for Unresectable Malignant Hilar Biliary Obstruction: A Single‐center Case Series (With Video)

**DOI:** 10.1002/deo2.70451

**Published:** 2026-10-13

**Authors:** Motoyasu Kan, Daiki Takahashi, Sodai Uchida, Taiga Sudo, Kouki Yokozuka, Yoshiki Nakaya, Akinori Abe, Hiroki Kurosaki, Yoshiki Ogane, Kazuki Watabe, Miho Sakai, Yu Sekine, Mayu Ouchi, Hiroshi Ohyama, Izumi Ohno, Jun Kato

**Affiliations:** ^1^ Department of Gastroenterology, Graduate School of Medicine Chiba University Chiba Japan; ^2^ Department of Oncology, Graduate School of Medicine Chiba University Chiba Japan

**Keywords:** inside stent, malignant hilar biliary obstruction, plastic stent, recurrent biliary obstruction, stent migration

## Abstract

**Background and Aims:**

Inside stents are increasingly used for malignant hilar biliary obstruction because of their prolonged patency and removability. Although reported migration rates vary, migration remains a clinical concern, and deployment in angulated bile ducts can be technically challenging. We evaluated a novel pig‐tail type inside stent (X‐FLEX ARC type) in unresectable malignant hilar biliary obstruction.

**Methods:**

This retrospective single‐center case series included consecutive patients who underwent X‐FLEX ARC type placement between October 2024 and December 2025. Procedural characteristics were evaluated for the 26 deployed ARC‐type stents, whereas clinical outcomes were evaluated after the 13 initial drainage procedures.

**Results:**

Eighteen patients underwent 21 endoscopic retrograde cholangiopancreatography sessions, during which 26 ARC‐type stents were deployed. Deployment into the intended bile duct branch was completed in all selected sessions. The median placement time was 4.0 min, and additional maneuvers were required for five stents (19.2%). Among 13 initial drainage procedures, clinical success was achieved in 11 (84.6%), and no procedure‐related adverse events occurred. Recurrent biliary obstruction (RBO) occurred after five procedures (38.5%), with a median time to RBO of 167 days (95% confidence interval, 137–not reached). No ARC‐type stent migration was observed.

**Conclusions:**

This preliminary case series provides descriptive information on ARC‐type inside stent placement and removability in selected patients with unresectable malignant hilar biliary obstruction. Prospective comparative studies are needed to determine its technical applicability and effects on stent migration and long‐term patency.

**Trial Registration**: N/A

**Abbreviations**
AE: adverse event
ARC: X‐FLEX ARC type inside stent
CI: confidence interval
ENBD: endoscopic nasobiliary drainage
ERCP: endoscopic retrograde cholangiopancreatography
IQR: interquartile range
RBO: recurrent biliary obstruction
SEMS: self‐expandable metal stent
TRBO: time to recurrent biliary obstruction

## 1 Introduction

Plastic stents are widely used for endoscopic biliary drainage in patients with malignant biliary obstruction because they are less expensive than self‐expandable metal stents (SEMSs) and can be removed and exchanged during reintervention. However, their relatively short patency and susceptibility to occlusion remain major limitations.

Inside stents are placed above the sphincter of Oddi to preserve sphincter function and reduce duodenobiliary reflux, potentially prolonging stent patency [1]. Initially reported for malignant hilar biliary obstruction in 2013 [2, 3], they have since shown lower recurrent biliary obstruction (RBO) rates and longer patency than transpapillary plastic stents in a meta‐analysis [4], and patency comparable to SEMSs in a Japanese randomized trial [5]. Their use has also expanded to preoperative biliary drainage and benign biliary strictures [6, 7].

Commercially available inside stents include straight, light‐angle, and deep‐angle configurations, allowing selection according to the target bile duct anatomy. Although reported migration rates vary among studies, a meta‐analysis found a migration rate of 13.2%, indicating that migration remains a relevant clinical concern [4]. In addition, placement in markedly angulated bile duct branches, such as the B3 segmental duct and posterior segmental branch, can remain technically challenging.

To address these technical considerations, a novel inside stent (X‐FLEX ARC type; Olympus Marketing, Inc., Tokyo, Japan) featuring a flexible pig‐tail distal end was developed with the aim of improving intrabiliary stability and facilitating placement in anatomically challenging bile duct branches. However, no clinical data regarding this device have been reported to date.

Therefore, the present study aimed to evaluate the feasibility, safety, and clinical outcomes of the novel X‐FLEX ARC inside stent in patients with unresectable malignant hilar biliary obstruction.

## 2 Materials and Methods

### 2.1 Study Design and Patients

This retrospective single‐center case series included consecutive patients with unresectable malignant hilar biliary obstruction who underwent X‐FLEX ARC type stent placement at our institution between October 2024 and December 2025. To describe drainage‐device selection, we also identified all patients who underwent endoscopic retrograde cholangiopancreatography (ERCP) for this condition during the study period, excluding balloon enteroscopy‐assisted ERCP in patients with surgically altered anatomy. Patients were classified as receiving an ARC‐type stent, receiving conventional inside stents without an ARC‐type stent, or receiving no inside stent. The study cohort comprised 18 patients undergoing 21 ERCP sessions.

### 2.2 Indications for the Novel Pig‐tail Inside Stent

The specific target bile duct branch and the type of inside stent were determined by the endoscopist during ERCP based on cholangiographic findings and the intended drainage strategy. The ARC type stent was preferentially selected when the target branch showed marked angulation on cholangiography or when stable positioning of a conventional straight‐type inside stent was considered potentially difficult. Such anatomical configurations were frequently encountered in the B3 segmental duct and posterior segmental branch; however, ARC stent selection was not based solely on the anatomical name of the target branch. No prespecified numerical threshold was used to define bile duct angulation. Because the specific target branch was determined during ERCP rather than predefined before the procedure, changes from a preprocedurally intended branch were not applicable.

### 2.3 Stent Characteristics and Endoscopic Procedure

The X‐FLEX ARC type is a newly developed 7‐Fr inside plastic stent available in lengths of 9 and 12 cm (Figure 1). The stent features a flexible pig‐tail distal end designed to improve intrabiliary stability. In addition, the transition between the guidewire, delivery catheter, and stent has been minimized to facilitate device advancement through angulated bile ducts. The retrieval lasso is positioned outside the shaft and therefore does not require deployment after stent placement. Similar to conventional inside stents, the stent can be removed by grasping the retrieval lasso during reintervention.

**FIGURE 1:**
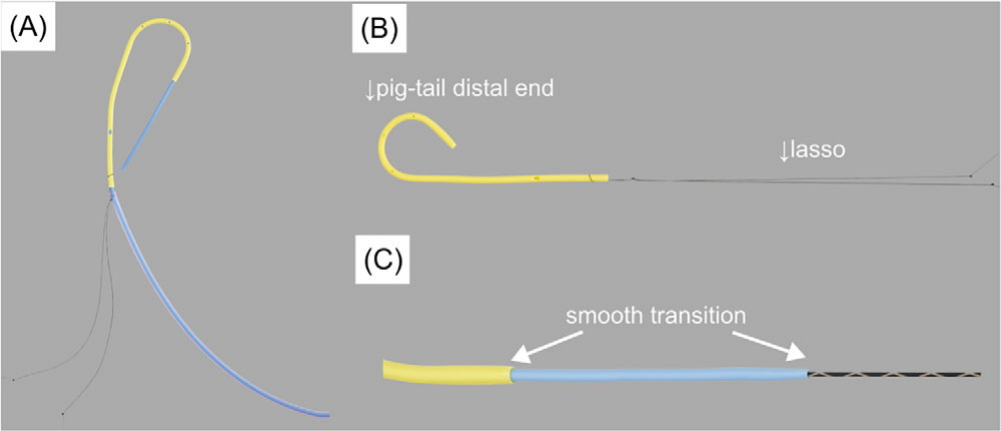
ARC type inside stent. (A) Overall appearance of the ARC type inside stent (Olympus Marketing, Inc., Tokyo, Japan). (B) Magnified view of the flexible pig‐tail distal end and external retrieval lasso. (C) Magnified view of the minimized transition between the stent, delivery catheter, and guidewire designed to facilitate advancement through angulated bile ducts.

All ERCP procedures were performed using standard techniques. The target bile duct branch was selectively cannulated using a guidewire, followed by deployment of the ARC‐type inside stent above the sphincter of Oddi.

### 2.4 Outcomes

Procedural experience with ARC‐type stent placement was evaluated descriptively using successful deployment into the intended bile duct branch, placement site, placement time, and delivery difficulty requiring additional maneuvers. Placement time was measured from confirmation of guidewire placement in the target branch to completion of ARC stent deployment, separately for each stent; time for additional maneuvers was included. Delivery difficulty was assessed for each deployed ARC type stent and was defined as failure of initial delivery‐system advancement requiring an unplanned corrective maneuver, including guidewire replacement, repeated delivery‐system advancement, or unplanned stricture dilation. Planned dilation before initial advancement was excluded. Clinical outcomes included clinical success, adverse events (AEs), stent migration, RBO, time to RBO (TRBO), stent removal, and reintervention.

Clinical success, AEs, RBO, and TRBO were defined according to the Tokyo Criteria 2024 [8]. TRBO was defined as the interval between stent placement and the occurrence of RBO. Patients without RBO were censored at the time of elective stent exchange, planned stent removal, surgery, death, or last follow‐up.

For procedural classification, initial drainage was defined as ARC type stent placement performed either during the initial biliary drainage procedure or as planned internalization following temporary endoscopic nasobiliary drainage (ENBD) during the same initial drainage episode. Sessions involving scheduled stent exchange or reintervention for RBO were classified as non‐initial drainage procedures. In patients who underwent internalization from ENBD, clinical success was considered achieved when biliary drainage was maintained or further improved after placement of the inside stent. Elective stent exchange sessions without jaundice or cholangitis were excluded from the evaluation of clinical success.

Stent migration was defined as displacement of the stent from its initial position requiring endoscopic reintervention and was classified as proximal migration or distal migration.

### 2.5 Data Collection

Patient demographics, underlying disease, Bismuth classification, target drainage branches, procedural details, clinical outcomes, and reintervention data were retrospectively collected from electronic medical records.

The study protocol was approved by the institutional review board of our hospital (approval number: HK202604‐12).

## 3 Results

### 3.1 Baseline and Procedural Characteristics

During the study period, 985 ERCP sessions were performed at our institution, including 142 sessions for malignant hilar biliary obstruction. Among these, 46 patients underwent 105 ERCP sessions for unresectable malignant hilar biliary obstruction. After excluding one patient who underwent two balloon enteroscopy‐assisted ERCP sessions, 45 patients undergoing 103 conventional ERCP sessions constituted the source population for assessment of drainage‐device selection. Of these 45 patients, 18 received an ARC‐type stent at least once, 14 received conventional inside stents without ARC‐type stent placement, and 13 were managed without inside stents. The latter group received other drainage devices, including SEMSs and transpapillary plastic stents.

The present case series included the 18 patients who received ARC type stents during 21 ERCP sessions (Table 1). The median age was 74 years (interquartile range [IQR], 70–78 years), and 14 patients (77.8%) were male. Gallbladder cancer, including postoperative recurrence and cystic duct cancer, was the most common underlying disease (7/18, 38.9%), followed by perihilar cholangiocarcinoma (6/18, 33.3%) and intrahepatic cholangiocarcinoma (4/18, 22.2%). Bismuth type III or higher lesions accounted for 17 of 21 ERCP sessions (81.0%).

**TABLE 1:** Baseline and procedural characteristics.

Variable	Overall
Number of patients	18
Number of ERCP sessions	21
Age, years, median [IQR]	74 [70–78]
Male sex, *n*/*N* (%)	14/18 (77.8)
Primary disease, *n* (%)	
Perihilar cholangiocarcinoma	6/18 (33.3)
Intrahepatic cholangiocarcinoma	4/18 (22.2)
Gallbladder cancer^*^	7/18 (38.9)
Distal bile duct cancer	1/18 (5.6)
Bismuth classification^†^, *n* (%)	
Type I	1/21 (4.8)
Type II	3/21 (14.3)
Type IIIa	10/21 (47.6)
Type IIIb	4/21 (19.0)
Type IV	3/21 (14.3)
Jaundice before drainage, *n*/*N* (%)	13/21 (61.9)
Cholangitis before drainage, *n*/*N* (%)	8/21 (38.1)
Initial drainage^§^, *n*/*N* (%)	13/21 (61.9)
Drainage area, *n* (%)	
Bilateral	11/21 (52.4)
Unilateral	10/21 (47.6)
Number of stents per session, *n* (%)	
1/2	10 (47.6) / 11 (52.4)
Both stents were ARC type^‡^, *n*/*N* (%)	5/11 (45.5)

Data are presented as *n*/*N* (%) or median [IQR].

Patient‐level variables were analyzed per patient, whereas procedure‐related variables were analyzed per ERCP session.

* Including postoperative recurrent gallbladder cancer and cystic duct cancer.

^†^ Calculated per ERCP session.

^‡^ Calculated among sessions with two stents placed.

^§^ Initial drainage included ARC type stent placement performed during the initial biliary drainage procedure or as planned internalization following temporary ENBD.

Jaundice and cholangitis were present in 13 (61.9%) and eight (38.1%) sessions, respectively. Initial biliary drainage was performed in 13 sessions (61.9%). Bilateral drainage was performed in 10 sessions (47.6%), and two stents were placed in 11 sessions (52.4%). Among these, bilateral ARC‐type stents were used in five of 11 sessions (45.5%).

### 3.2 Stent Characteristics and Clinical Outcomes

A total of 26 ARC‐type stents were deployed (Table 2), most commonly in B3 (10/26, 38.5%) and the posterior branch (8/26, 30.8%); 16 (61.5%) were 9 cm and 10 (38.5%) were 12 cm. Fifteen sessions were ARC‐only and six were mixed ARC/conventional‐stent sessions. In mixed sessions, ARC stents were placed in the posterior branch (*n* = 3), B2 (*n* = 2), or B3 (*n* = 1), whereas conventional straight‐type stents were placed in B2 (*n* = 3) or the anterior branch (*n* = 3). Branch‐specific reasons for conventional‐stent selection could not be determined retrospectively; selection reflected cholangiographic anatomy and the intended drainage strategy. Thirteen sessions involved initial drainage, including planned internalization after temporary ENBD; the remaining eight comprised five RBO reinterventions and three scheduled exchanges.

**TABLE 2:** Procedural characteristics of the 26 ARC type inside stents.

Variable	Overall
Number of ARC type stents	26
Placement site, *n* (%)	
B2	4 (15.4)
B3	10 (38.5)
Anterior segmental branch (Ba)	4 (15.4)
Posterior segmental branch (Bp)	8 (30.8)
Stent length, *n* (%)	
9 cm	16 (61.5)
12 cm	10 (38.5)
ARC stent placement time, min, median (IQR)^a^	4.0 (3.0–7.8)
Additional maneuvers required, *n*/*N* (%)^b^	5/26 (19.2)
Repeated advancement of the delivery system	2/26 (7.7)
Unplanned biliary stricture dilation	2/26 (7.7)
Guidewire replacement	1/26 (3.8)

^a^ ARC stent placement time was measured from confirmation of guidewire placement in the target bile duct branch to completion of ARC stent deployment.

^b^ Additional maneuvers were evaluated for each deployed ARC type stent and included repeated advancement of the delivery system, unplanned biliary stricture dilation, and guidewire replacement.

IQR, interquartile range.

The median ARC stent placement time was 4.0 min (IQR, 3.0–7.8). Additional maneuvers were required for five of the 26 ARC type stents (19.2%). These consisted of repeated advancement of the delivery system for two stents, unplanned biliary stricture dilation for two stents, and guidewire replacement for one stent.

Clinical outcomes after the 13 initial drainage procedures are summarized in Table 3. Among these selected procedures, deployment of the ARC‐type stent into the intended bile duct branch was completed in all sessions. Clinical success was achieved in 11 sessions (84.6%), including nine of 10 ARC‐only sessions (90.0%) and two of three mixed sessions (66.7%). No procedure‐related AEs were observed.

**TABLE 3:** Clinical outcomes after initial biliary drainage.

Outcome	Overall	ARC‐only^*^	Mixed^*^
Number of initial drainage procedures	13	10	3
Successful deployment into the intended bile duct branch	13/13 (100.0)	10/10 (100.0)	3/3 (100.0)
Clinical success	11/13 (84.6)	9/10 (90.0)	2/3 (66.7)
Procedure‐related adverse events	0/13 (0.0)	0/10 (0.0)	0/3 (0.0)
Recurrent biliary obstruction	5/13 (38.5)	3/10 (30.0)	2/3 (66.7)
Causes of RBO^†^			
Stent occlusion	4/5 (80.0)	3/3 (100)	1/2 (50.0)
Migration of a concomitant conventional straight‐type stent^‡^	1/5 (20.0)	0/3 (0.0)	1/2 (50.0)
ARC type stent migration	0/13 (0.0)	0/10 (0.0)	0/3 (0.0)
TRBO, median days (95% CI)^§^	167 (137–NR)	—	—

Data are presented as *n*/*N* (%) unless otherwise indicated.

* ARC‐only sessions involved placement of ARC‐type stents alone, whereas mixed sessions involved concomitant placement of an ARC‐type stent and a conventional straight‐type inside stent.

^†^ Percentages for the causes of RBO were calculated using the number of sessions with RBO in each group as the denominator (overall, *n* = 5; ARC‐only, *n* = 3; mixed, *n* = 2).

^‡^ The migration‐related RBO occurred in a mixed session and involved the concomitantly placed conventional straight‐type inside stent, not the ARC‐type stent.

^§^ TRBO was estimated for the overall cohort of 13 initial drainage procedures using the Kaplan–Meier method. Subgroup‐specific estimates were not presented because of the small subgroup sizes and the descriptive nature of the study.

CI, confidence interval; RBO, recurrent biliary obstruction; TRBO, time to recurrent biliary obstruction; NR, not reached.

Among the 13 initial drainage sessions, RBO occurred in five sessions (38.5%), including three of 10 ARC‐only sessions (30.0%) and two of three mixed sessions (66.7%). Stent occlusion accounted for four of the five RBO events (80.0%). The remaining event occurred in a mixed session and was caused by migration of a concomitantly placed conventional straight‐type inside stent. No migration of an ARC‐type stent was observed. The median TRBO was 167 days (95% confidence interval [CI], 137–NR) in the overall initial drainage cohort (Figure 2).

**FIGURE 2:**
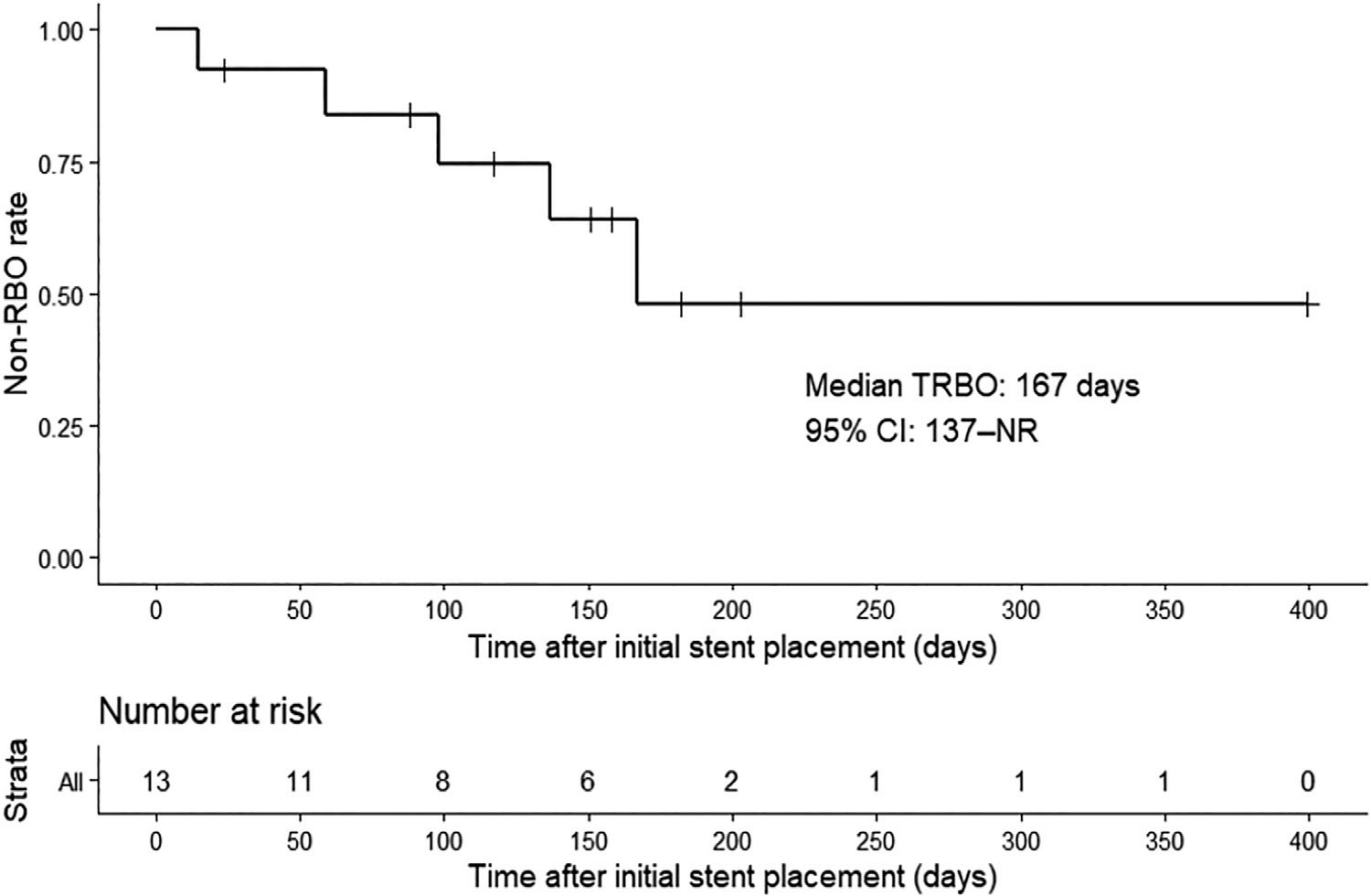
Kaplan–Meier analysis of time to recurrent biliary obstruction after initial biliary drainage. Kaplan–Meier curve showing time to recurrent biliary obstruction (TRBO) after the 13 initial drainage procedures, including 10 ARC‐only sessions and three mixed sessions. This curve represents an overall session‐level analysis and should not be interpreted as an ARC‐type stent‐specific patency curve. Tick marks indicate censored observations. NR, not reached.

Stent removal was attempted in nine sessions and was successful in all cases. Reintervention was performed in 8 sessions (38.1%), and all procedures were completed successfully.

### 3.3 Representative Cases

#### 3.3.1 Case 1

A patient with unresectable malignant hilar biliary obstruction required drainage of the posterior segmental branch. The novel pig‐tail type inside stent was successfully deployed into the target branch without the need for additional maneuvers (Figure 3 and Video S1).

**FIGURE 3:**
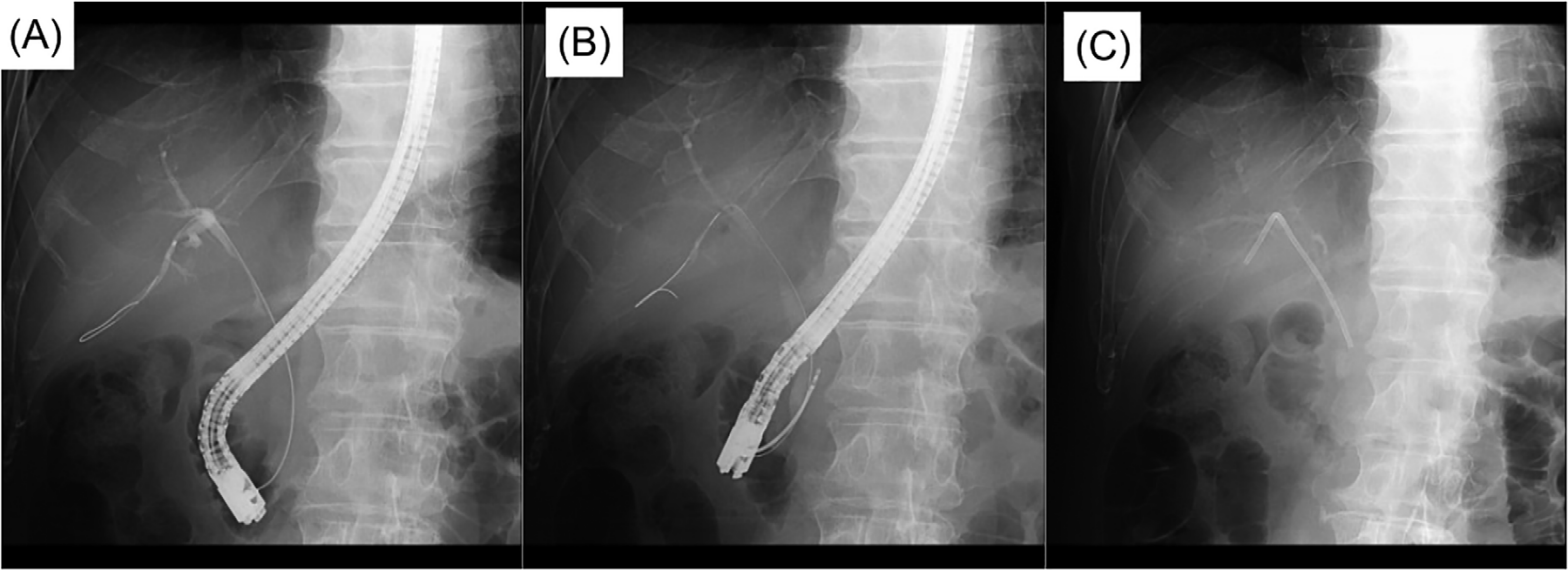
Representative case of ARC‐type stent placement in an angulated posterior branch. (A) Cholangiogram demonstrating malignant hilar biliary obstruction with involvement of an angulated posterior branch. (B) Advancement of the guidewire and delivery system into the target branch. (C) Successful deployment of the ARC‐type inside stent with stable positioning in the posterior branch.

#### 3.3.2 Case 2

In another patient, RBO developed because of proximal migration of a previously placed conventional straight‐type inside stent, necessitating reintervention. During reintervention, bilateral drainage was re‐established using ARC‐type inside stents on both sides (Figure 4).

**FIGURE 4:**
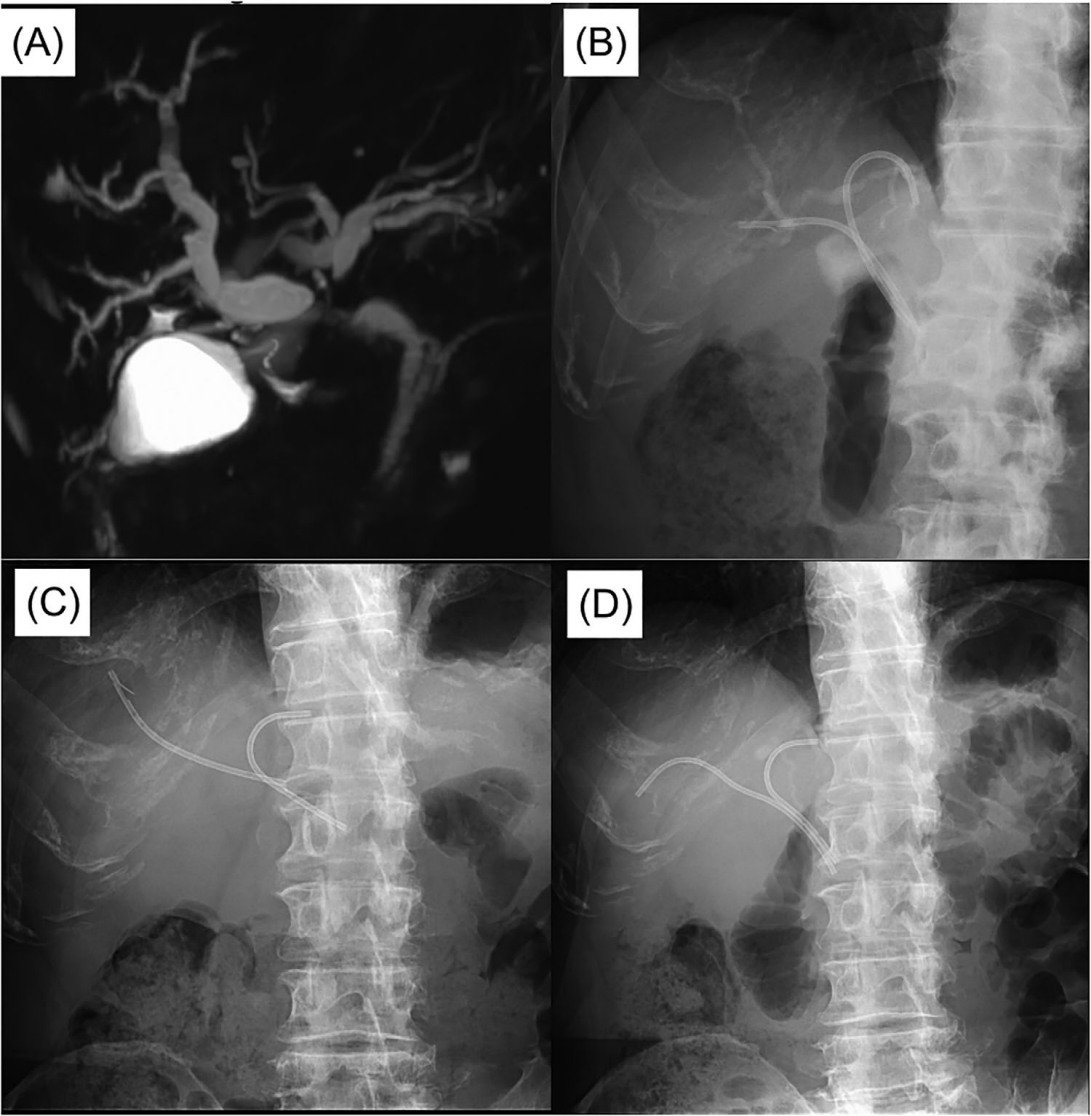
Representative case of migration associated with a conventional straight‐type inside stent. (A) MRCP demonstrating unresectable malignant hilar biliary obstruction (Bismuth type IIIa). (B) Bilateral drainage using a conventional straight‐type inside stent in the right hepatic duct and an ARC type inside stent in the left hepatic duct. (C) Recurrent biliary obstruction caused by migration of the conventional straight‐type inside stent. (D) Bilateral ARC‐type inside stents successfully deployed following reintervention.

## 4 Discussion

In the present study, we evaluated the initial clinical outcomes of a novel pig‐tail type inside stent in patients with unresectable malignant hilar biliary obstruction. Among the sessions in which the endoscopist judged the ARC type stent suitable on the basis of cholangiographic anatomy and the intended drainage strategy, deployment into the intended bile duct branch was completed in all 21 sessions. The median ARC stent placement time was 4.0 min, and additional maneuvers were required for five of the 26 deployed stents (19.2%). Among the 13 initial drainage procedures, no procedure‐related AEs were observed, while stent removal was successful in all nine attempted sessions. These observations are limited to a selected case series and should not be interpreted as evidence of general technical feasibility or superiority over conventional inside stents. Although no migration of the ARC‐type stent was observed, the small sample size and absence of a comparator group preclude conclusions regarding migration prevention or long‐term patency.

To the best of our knowledge, this is the first case series describing the clinical use of the ARC‐type inside stent. The present findings provide preliminary descriptive data on ARC type stent placement and removability in selected patients with unresectable malignant hilar biliary obstruction.

The most distinctive feature of this stent is its potential to improve intrabiliary stability. Although no stent migration was observed in the randomized trial by Kanno et al., reported migration rates vary among studies, and a meta‐analysis found migration in 13.2% of patients treated with inside stents [4, 5]. Migration may cause RBO and necessitate unplanned reintervention. In particular, proximal migration can make endoscopic stent retrieval technically challenging and occasionally unsuccessful [9]. Therefore, despite the low migration rate reported in some studies, there remains a clinical rationale for exploring stent designs that may improve intrabiliary stability. In the present study, no migration of the ARC‐type stent was observed; however, given the limited sample size and lack of a comparator group, no conclusion regarding migration prevention can be drawn. The pig‐tail distal end may function as an intrabiliary anchor, reducing axial displacement and thereby potentially decreasing the risk of migration. Further studies are required to determine whether this design translates into a clinically meaningful reduction in migration rates.

Another notable finding was that 69.2% of the deployed stents were placed in either the B3 segmental duct or posterior segmental branch. These bile duct branches are frequently associated with anatomical angulation and may require additional technical maneuvers during stent placement [10]. In the present study, ARC type stents were successfully deployed in selected branches, including the B3 segmental duct and posterior segmental branch. However, because the target branch and stent type were selected at the discretion of the endoscopist and bile duct angulation was not objectively quantified, these findings should be interpreted as descriptive procedural observations rather than evidence of an advantage in anatomically angulated bile ducts.

Conventional inside stents are also available in light‐angle and deep‐angle configurations, allowing selection according to the target bile duct anatomy. In particular, deep‐angle stents may conform well to strongly curved branches, including the posterior segmental branch, and may be more appropriate than a pig‐tail‐type stent in some anatomical configurations, as reported by Mori et al. [11]. Conversely, the flexible pig‐tail configuration of the ARC type may represent an alternative when stable placement of a conventional straight‐ or angle‐type stent is considered difficult. However, the present study does not establish an objective angle‐based selection criterion or demonstrate superiority of one configuration over another. Stent selection should therefore be individualized according to cholangiographic anatomy and the intended drainage strategy.

Among the 13 initial drainage procedures, the median TRBO was 167 days (95% CI, 137–NR). Although this value can be descriptively considered in the context of previously reported outcomes for inside stents [4], differences in patient characteristics, drainage strategies, and outcome assessment preclude direct comparisons across studies. Furthermore, the limited sample size, short follow‐up period, and inclusion of mixed drainage sessions prevent definitive evaluation of long‐term patency. Larger prospective studies are required to characterize RBO and TRBO after ARC type stent placement.

Taken together, the present findings provide preliminary information regarding ARC type stent placement and subsequent management. However, they do not establish comparative efficacy, superiority in anatomically challenging branches, or improved long‐term outcomes relative to conventional inside stents. RBO nevertheless occurred in a substantial proportion of sessions, underscoring the need for further evaluation of patient selection and drainage strategies.

An additional clinically important finding was that stent removal was attempted in nine sessions and was successful in all cases. This finding suggests that the ARC type stent preserves one of the major advantages of conventional inside stents: removability during reintervention. Advances in systemic chemotherapy and multidisciplinary treatment strategies have prolonged survival in patients with unresectable malignant hilar biliary obstruction, increasing the need for repeated biliary interventions during long‐term follow‐up. Furthermore, the concept of stent‐demanding time has recently been proposed as a clinically relevant outcome measure, emphasizing not only prolonged stent patency but also the importance of maintaining quality of life through effective and manageable biliary drainage strategies [8]. In a 25‐year experience involving patients with hilar and perihilar cholangiocarcinoma, plastic stents facilitated subsequent percutaneous transhepatic biliary drainage because of their removability, and double‐pigtail plastic stents were associated with fewer stent‐related AEs than straight‐type stents [12]. Furthermore, Sakai et al. suggested that the pig‐tail configuration conforms more naturally to the anatomy of the intrahepatic bile duct and may also reduce the risk of stent migration [13]. Although the present study was not designed to evaluate migration prevention, reintervention frequency, or long‐term efficacy, successful removal in all attempted sessions provides preliminary evidence that the ARC type stent retains the removability expected of an inside plastic stent.

This study has several limitations. First, it was a retrospective single‐center study with a relatively small sample size. Second, the target bile duct branch and stent type were selected at the discretion of the endoscopist during ERCP, and bile duct angulation was not assessed using a prespecified objective threshold. Therefore, selection bias cannot be excluded. Third, no direct comparison with conventional straight‐type inside stents was performed; therefore, the present study cannot determine whether the ARC type stent facilitates placement or reduces the risk of migration compared with conventional devices. Finally, the follow‐up period was limited. Therefore, larger prospective studies and comparative investigations are required to further clarify the clinical role of this novel device.

## 5 Conclusion

In conclusion, this small retrospective case series provides preliminary descriptive information on ARC type inside stent placement and removability in selected patients with unresectable malignant hilar biliary obstruction. Prospective comparative studies are needed to determine its technical applicability and effects on procedural difficulty, stent migration, and long‐term patency.

## Author Contributions

Motoyasu Kan conceived and designed the study, collected the data, and conducted the statistical analysis. MK drafted the manuscript. All authors (Motoyasu Kan, Daiki Takahashi, Sodai Uchida, Taiga Sudo, Kouki Yokozuka, Yoshiki Nakaya, Akinori Abe, Hiroki Kurosaki, Yoshiki Ogane, Kazuki Watabe, Miho Sakai, Yu Sekine, Mayu Ouchi, Hiroshi Ohyama, Izumi Ohno, and Jun Kato) critically reviewed the manuscript for important intellectual content and approved the final version for submission.

## Funding

The authors have nothing to report.

## Ethics Statement

This retrospective study was approved by the institutional review board of Chiba University Hospital (approval number: HK202604‐12). The need for informed consent was waived owing to the retrospective design of the study, with data anonymized prior to analysis. The study was conducted in accordance with the Declaration of Helsinki.

## Conflicts of Interest

Motoyasu Kan was involved in the development of the X‐FLEX ARC type stent and participated in its pre‐commercial clinical use. He received no financial compensation related to the device. The other authors declare no conflicts of interest.

## Supporting information




**Video S1**: Representative placement of the ARC type inside stent in an angulated posterior branch. The video demonstrates guidewire advancement into the angulated posterior branch, delivery of the ARC type inside stent, and successful deployment with stable intraductal positioning in a patient with malignant hilar biliary obstruction.
